# Comparative gene expression between two yeast species

**DOI:** 10.1186/1471-2164-14-33

**Published:** 2013-01-16

**Authors:** Yuanfang Guan, Maitreya J Dunham, Olga G Troyanskaya, Amy A Caudy

**Affiliations:** 1 Lewis-Sigler Institute for Integrative Genomics, Princeton University, Princeton, NJ 08544, USA; 2 Department of Molecular Biology, Princeton University, Princeton, NJ 08544, USA; 3 Current Address: Department of Computational Medicine & Bioinformatics, University of Michigan, Ann Arbor, MI 48109, USA; 4 Department of Genome Sciences, University of Washington, Seattle, WA 98195, USA; 5 Department of Computer Science, Princeton University, Princeton, NJ 08540, USA; 6 Donnelly Centre for Cellular and Biomolecular Research, University of Toronto, Toronto, Ontario M5S 3E1, Canada

**Keywords:** Expression divergence, Yeast, Microarray, Data integration, Condition-specific gene expression

## Abstract

**Background:**

Comparative genomics brings insight into sequence evolution, but even more may be learned by coupling sequence analyses with experimental tests of gene function and regulation. However, the reliability of such comparisons is often limited by biased sampling of expression conditions and incomplete knowledge of gene functions across species. To address these challenges, we previously systematically generated expression profiles in *Saccharomyces bayanus* to maximize functional coverage as compared to an existing *Saccharomyces cerevisiae* data repository.

**Results:**

In this paper, we take advantage of these two data repositories to compare patterns of ortholog expression in a wide variety of conditions. First, we developed a scalable metric for expression divergence that enabled us to detect a significant correlation between sequence and expression conservation on the global level, which previous smaller-scale expression studies failed to detect. Despite this global conservation trend, between-species gene expression neighborhoods were less well-conserved than within-species comparisons across different environmental perturbations, and approximately 4% of orthologs exhibited a significant change in co-expression partners. Furthermore, our analysis of matched perturbations collected in both species (such as diauxic shift and cell cycle synchrony) demonstrated that approximately a quarter of orthologs exhibit condition-specific expression pattern differences.

**Conclusions:**

Taken together, these analyses provide a global view of gene expression patterns between two species, both in terms of the conditions and timing of a gene's expression as well as co-expression partners. Our results provide testable hypotheses that will direct future experiments to determine how these changes may be specified in the genome.

## Background

The Ascomycete yeasts present one of the most promising systems for comparative functional genomics. Fungi have been densely sampled by a number of sequencing projects [1], covering an enormous range of divergence. Genome sequence analyses of the *Saccharomyces* yeasts and related species have been used to establish the history of gene duplication [2-6], conservation at binding sites [7,8], and co-evolution of binding sites with regulators [9]. Thus, a range of evolutionary phenomena can be studied in these species based on their genomic sequence. However, sequence conservation is not always completely predictive of functional conservation. As just one example, we recently reported that only a subset of conserved promoter motifs actually drive periodic gene expression over the cell cycle in two closely related species [10].

Most of the experimental characterization of gene function has been performed in a small number of model fungal systems, which can provide an anchor for these broad genome sequencing surveys. These species include *Saccharomyces cerevisiae*, *Neurospora crassa, Candida albicans*, and *Schizosaccharomyces pombe*, along with several other emerging models such as *Ashbya gossypii*. Comparative studies between these species, which by some estimates cover a billion years of divergence, have been informative [11,12]. Analysis of gene expression changes over growth [13], the cell cycle [13], and stress treatments [14,15] highlighted both similarities and differences in ortholog expression. Unfortunately, the ability to link individual gene expression divergence with the causative molecular factors has been limited because of the vast evolutionary distances involved.

Experimental protocols developed in the model systems are often readily portable to less well-studied sister species, allowing us to choose species well-placed to identify and study functional divergence. Comparisons of gene expression across particular species with interesting characteristics can not only highlight how patterns of gene expression change over evolutionary time, but can also discover genes with particular functions. A comparison among xylose-metabolizing species of yeasts, for example, was able to couple sequence analysis with gene expression profiling to identify important genes via their presence in the genomes of interest and their induction when grown on xylose [16]. Followup studies in *S. cerevisiae* confirmed these associations.

Due to their close proximity to *S. cerevisiae,* studies in the *sensu stricto* yeasts have also been particularly informative. These species cover a range of conservation, have high quality annotated reference genomes [17], and are becoming even more attractive as the sequences of many strains within each species are forthcoming using new high-throughput sequencing tools (e.g. [18]). Furthermore, their ability to form interspecific hybrids leverages the resources available in *S. cerevisiae* and allows tests of gene function and regulation in shared cellular environments [19-23]. Recent work on expression-based full-genome characterization is reported in [24,25], which used *S. cerevisiae* microarrays to measure the gene expression consequences of heat shock stress and mating induction on three other yeast species. Their data suggest that expression divergence can occur relatively rapidly and is correlated to gene function, though relatively uncorrelated to sequence conservation [26]. Due to the *S. cerevisiae* arrays used, they were unable to examine more divergent species. In order to broaden these studies to more divergent yeasts, species-specific arrays must be used, as has been done, for example, for *Candida glabrata*[27]. Most importantly, due to the limited condition space of just a small number of treatments in these studies, conclusions about evolution of gene function and regulation have been difficult to generalize.

To address these challenges, we previously developed a computational framework [28,29] to identify a set of experiments that could best characterize gene function in a naive species. Based on available expression date in the *S. cerevisiae* literature, we identified and carried out a set of 304 experiments over 46 conditions in the *sensu stricto* species *S. bayanus var uvarum.* By choosing only the most informative experiments from the vast *S. cerevisiae* literature, we were able to survey a large phenotypic space at high accuracy with a modest amount of experimentation.

To compare these expression datasets more carefully, we developed a statistical metric, Local Network Similarity (LNS), to assess correlation patterns of orthologs. This metric is general and robust – it can be used for analysis of individual matched datasets without the need to assume identical response time for the two species, or for integrated analysis of diverse compendia of experimental or genetic perturbations. Using the LNS metric to compare our large *S. bayanus* expression compendium with a collection of published *S. cerevisiae* expression data, we show that gene expression networks are largely conserved between the species, though much less than within-species comparisons constructed by comparing different conditions. Furthermore, we demonstrated strong and statistically significant evidence for correlation between the divergence of expression and open reading frame sequence, which previous studies using more limited datasets failed to detect (see review [26]). Despite this general conservation pattern, we observed that a quarter of orthologs exhibit condition-specific differences in expression, and 4% show strong differences in global co-expression patterns. Genes involved in the same functional groups share similar divergence patterns, indicating that pathways or processes may share characteristics. In sum, our wide-ranging survey of expression profiles and generic metric of expression divergence allowed us to identify both global and local aspects of regulatory evolution and relate these to sequence divergence.

## Results

*S. bayanus var uvarum* (referred to as “*S. bayanus*” from here forward) is a sequenced but relatively unstudied species of budding yeast typically associated with fermentation environments and diverged by approximately 20 million years from *S. cerevisiae*. We have recently computationally chosen 46 biological treatments for gene expression analysis that would maximally cover biological processes in yeast [28]. Using the resulting dataset of over 300 arrays in *S. bayanus* along with 2569 arrays collected from *S. cerevisiae*[30-34], we carried out both global and dataset-specific comparisons of gene expression.

### A generic statistical metric to quantify expression conservation between species

To measure the divergence in gene expression between the two species, we developed a metric to compare the expression networks surrounding orthologous genes. Since gene co-expression is strongly linked to functional similarities, differences in expression neighborhoods over a reasonably comprehensive set of perturbations provide a robust proxy of functional relationships. Previously developed methods of analyzing co-expression patterns across species have relied on producing matched datasets, in which comparable timepoints were collected from multiple species exposed to the same conditions [24]. However, exactly matched datasets are difficult to gather, and such an approach relies on the assumption that the precise timing of expression change should be conserved. We sought to develop a method that could detect large-scale patterns of co-expression in addition to those found under specifically matched conditions.

**Figure 1:**
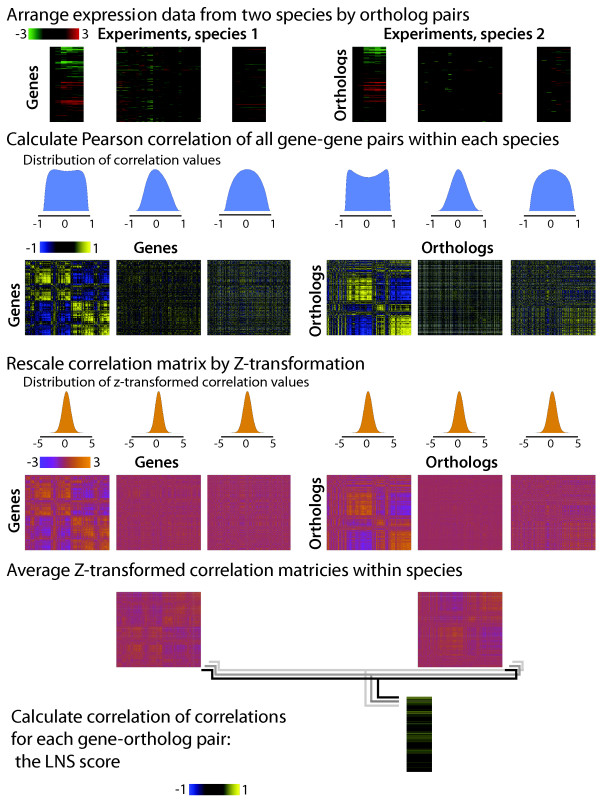
**Quantification of expression conservation by local network similarity (LNS).** Pair-wise Pearson correlation between genes was calculated for individual *S. bayanus* and *S. cerevisiae* datasets, generating a matrix of gene-gene correlations. The data used to create this illustration are the actual diauxic shift, cell cycle synchronization, and alpha factor treatment. The distribution of these correlation values is between −1 and 1, and can be drastically different from one dataset to another. Therefore, Fisher’s z-transformation and normalization of these z-values were applied on each correlation matrix, so that the correlations were comparable across datasets. The resulting correlation matrices are normally distributed and centered at 0 with standard deviation equal to 1. For each orthologous pair *i* and *i’*, their z-transformed, normalized correlation to all other matched orthologs form two vectors, indicating the relative positions of this pair of ortholog in their respective expression network. The correlation of these two vectors was taken as LNS. To calculate the correlation matrix for global LNS, the average values of individual datasets for a specific gene-gene pair were used to form a new global correlation matrix. According to the properties of normal distribution, the values within this matrix are still normally distributed and centered at 0 with standard deviation equal to 1. This global matrix was then used to calculate global LNS for each ortholog. To simulate the case of non-conservation, orthologous pairs were randomized along one axis of the expression correlation matrix. Therefore in calculating background LNS, only the ortholog match was disturbed, but not the expression network structure (in contrast to randomizing along both axes).

In order to quantify expression divergence at this global level, we developed a metric, Local Network Similarity (LNS), which measures the similarity of expression connections around each member of an ortholog pair (Figure 1). This metric is conceptually inspired by previous analyses that summarize the entire network of co-expression that exists between a gene and the rest of the genes in the genome [35,36]. The distribution of correlation coefficients of different datasets varies greatly, as is demonstrated in Figure 1, which plots actual expression datasets from the two species. LNS is calculated by stabilizing the variance of the distribution of correlation coefficients by first normalizing the within-species gene-gene correlation coefficients using the Fisher transformation and then further normalizing these data to the standard normal distribution. This normalization prevents domination by few changes of large magnitude or loss of signal from changes that occur in only a subset of perturbations (see Methods for details). The expression conservation of a pair of orthologous genes is thus quantified based on the preservation of all the expression connections around the pair of orthologs, i.e., the similarity of the local, first-degree expression networks around the two genes. The distribution of LNS of a completely non-conserved network resembles the normal distribution, making direct hypothesis tests possible.

Notably, and in contrast to previous global comparison approaches, this definition does not rely on alignment of individual datasets but defines a gene’s expression pattern in the context of the genome-wide co-expression network. Therefore, the LNS concept could be extended to integrate any number of genome-scale expression datasets—or even other types of genomic data—to quantify conservation between any two species pairs.

### Global expression conservation and divergence identified by LNS

**Figure 2:**
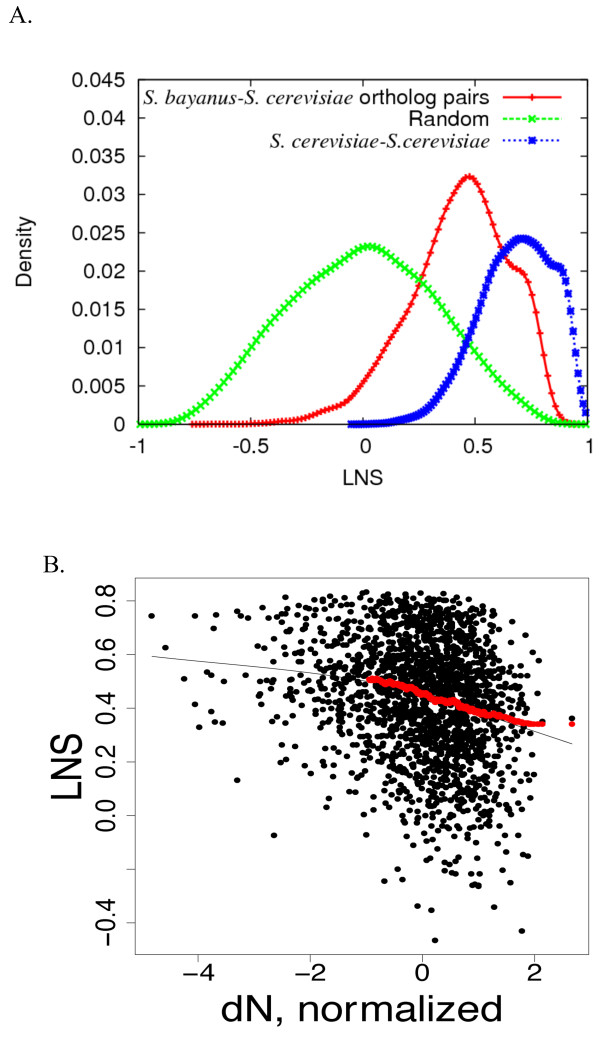
**Expression conservation relationship with sequence conservation. A**. Compared to randomized gene pairs, distribution of LNS scores of all one-to-one ortholog pairs is shifted towards positive values, though this distribution is less positive than comparisons within species. **B**. LNS is correlated with sequence divergence with a Pearson correlation of −0.235 (p<2.2e-16). Black line is loess smoothed curve line and red line is running average of LNS summed over 100-gene windows. Graphs for other measures of sequence divergence are in Figure S2.

LNS revealed considerable conservation of correlation structure between the two species’ expression networks. We simulated the case of zero conservation by randomizing the orthologous pairs while preserving the network structure (Figure 1). This simulation resulted in LNS scores normally distributed and centered at 0 (Figure 2A). Unlike the randomized network, the LNS scores based on the matched orthologs were distributed from −0.63 to 0.83 with the median of 0.45 (Figure 2A, Additional file 1: Table S1), showing a clear shift towards positive values. This demonstrates that on average, orthologs preserved their expression network. A right shoulder in the LNS distribution suggests that there is a subset of genes with very highly conserved coexpression networks; this population of genes with high LNS persists even when ribosomal genes are removed from the data (data not shown).

In order to test whether this conservation is more or less than what would be expected at this degree of sequence divergence, we would ideally compare a similar gene expression compendium collected from genetically diverse isolates within each species or in species at different genetic distances. Although some data exist exploring differences in gene expression among *S. cerevisiae* strains [37-39], there are not sufficient datasets available for a full test. However, we were able to test the limit case of LNS as compared between different subsets of the *S. cerevisiae* literature. These experiments survey a wide diversity of conditions and have been performed in different strain backgrounds, though mostly focused on a small number of related lab strains. The LNS distribution within the subsampled *S. cerevisiae* datasets was significantly more positive than both the random distribution and the between species comparison (Figure 2A, Additional file 2: Table S2). This result demonstrates the robustness of LNS for identifying patterns of co-expression even across very different environmental and genetic conditions, and suggests that the level of coexpression difference observed between *S. cerevisiae* and *S. bayanus* is probably beyond what is present within a species.

Cross-species LNS revealed some dramatic changes in correlation patterns in addition to the overall pattern of conservation. We found 183 genes out of the 4701 orthologous pairs (4%) with an LNS lower than 0, suggesting these genes changed globally in their expression network (Additional file 1: Table S1). The orthologs with low LNS represent several underlying biological causes. One obvious category is genes known to carry deficiencies in laboratory strains. For example, the promoter of *CTR3* is disrupted by a Ty2 insertion in many lab strains of *S. cerevisiae*[40], but is intact in *S. bayanus*. Most of the data in the *S. cerevisiae* compendium is derived from lab strains in which *CTR3* expression is driven by the transposon promoter, while the native promoter is present in the *S. bayanus* strains used in our compendium, leading to very different expression patterns and a low LNS score of −0.07166.

Similarly, we noted a number of targets of alpha factor signaling among the lowest LNS scores, and subsequently examined a list of known transcriptional targets of signaling through Gpa1, the alpha component of the heterotrimeric G protein that activates the MAP kinase cascade in yeast [41]. The median LNS of the *GPA1* targets is 0.27, significantly lower than the set of all genes. S288c derived laboratory strains of *S. cerevisiae* carry a S469I *GPA1* mutation that increases signaling through the MAP kinase cascade, but *S. bayanus* and all other members of the *sensu stricto* group carry the ancestral allele [42]. Therefore, the low overall LNS scores of the *GPA1* target genes may reflect the difference between wild type *GPA1* activity in *S. bayanus* and hyperactive signaling in laboratory strains of *S. cerevisiae*. Background mutations in *S. bayanus* can be identified as well, including the alpha factor protease gene [43], *BAR1*, which is mutant in the *S. bayanus* strain used in nearly every experiment in our expression compendium, and scored a low 0.26 LNS.

The second category of low LNS scores represents incorrectly annotated ortholog pairs. In our initial analysis, we discovered a number of cases of improperly assigned orthologs (Table 1). For instance, the *S. bayanus* gene *620.38* had been assigned the ortholog *RAS1* during the initial annotation effort [8], and had an LNS of −0.38. However, the protein sequence of the *620.38* ORF has only partial homology to Ras1 and is in fact a close homolog of the vacuolar protein Vps21. In addition, *620.38* is syntenic with *VPS21*[44]. This example demonstrates that LNS provides a functional criterion for ortholog identification and validation.

**Table 1:** Mis-annotated genes identified by LNS

**Gene**	**Old ortholog**	**New ortholog**	**LNS**	**Comments**
***620.38***	***YOR101W***	***YOR089C***	**−0.379**	**Blast e-value to *VPS21 *is 1e-92. Blast to *RAS1 *is 11th on list.**
***576.11***	***YGL157W***	***YGL039W***	**0.184**	**Note similar synteny conflict with *674.45*. This gene is second best blast, e-value 1e-160.**
***674.45***	***YGL039W***	***YGL157W***	**−0.077**	**Synteny preserved by change, new gene is best blast hit with e-value 3e-146. Note similar problem with *576.11*.**
***635.17***	***YOR267C***	***YOR233W***	**−0.633**	**Synteny preserved by change. *YOR233W *is best blast hit with e-value 0.**
***636.13***	***YPR119W***	***YPR120C***	**−0.03**	
**(cell cycle)**	**Best hit with *CLB5 (YPR120C)*.**			

Ortholog pairings were initially taken from the original genome annotation [7]. Several mis-pairs were identified by their low global or condition-specific LNS.

The remaining genes with low LNS are from diverse biological processes and functions. These genes are enriched for orthologs whose *S. cerevisiae* annotations include genes involved in ascospore cell wall formation (GO:0009272 fungal cell wall-type biogenesis, Bonferroni-corrected p-value 8.97x10^^-5^ and related terms), and other developmental processes involved in mating and meiotic division, leading to the intriguing hypothesis that gene expression network differences may be related to speciation between these two yeasts. However, genes associated with these biological processes accounted for only 15% of the highly divergent genes. Multiple genes in this set are associated with DNA synthesis and repair, signaling, chromatin organization, metabolism, and transcription, among many other processes, emphasizing that differences are present throughout the cellular network. This list of low LNS genes in known functions should assist the prioritization of experimental work to determine the basis of evolutionary changes in expression. A full quarter of the genes with low LNS scores are of unknown function. Further experiments will be required to determine the mechanisms by which these genes diverge in their expression networks, and the degree to which these differences may contribute to phenotypic differences between the species.

### Sequence conservation predicts gene expression divergence

Despite the requirement for experimental followup of individual ortholog pairs, LNS analysis on our large data collection allows us to test several hypotheses regarding the overall role of genome sequence in determining interspecies variation of gene expression. First, we considered the effect of promoter structure by grouping ortholog pairs into TATA-containing in both species, TATA-containing in one of the members, and TATA-less. Though not statistically significant (Additional file 3: Figure S1, *r* = −0.02, *p* = 0.08), TATA-containing orthologs have lower LNS, indicating higher interspecies variation, consistent with previous results [24] using other yeast species and measurements of expression divergence.

Secondly, changes in promoter sequence could potentially cause changes in gene expression, so we extended the evaluation of promoters to examine the relationship between upstream sequence conservation and local network similarity. Upstream sequence conservation is weakly predictive of expression conservation (Additional file 3: Figure S1, *r* = 0.047, *p* = 0.00016, *n* = 4701).

Thirdly, in contrast to the small effects of TATA promoter type or upstream sequence conservation, we found a stronger correlation (Figure 2B, *r* = −0.235, *p* < 2.2e-16) between protein sequence similarity and local network similarity. This relationship was observed when using several different methods of calculating sequence similarity (Additional file 3: Figure S2). This correlation between protein sequence and expression similarity is consistent with the majority of results in mammalian systems [45-47], *Xenopus*[48] and *Drosophila*[49,50]. This result contrasts with the conclusions of previous studies in yeast that did not detect any relationship between sequence conservation and expression conservation [26]. Our ability to detect this relationship may result from our use of a large, diverse expression compendium and a more generic measurement of expression divergence. Indeed, if we focus solely on a pair of cell cycle datasets and align them by time points, similar to previous works [24,25], we do not detect correlation between sequence conservation and expression conservation (not shown). As a result, although using aligned datasets could help identify orthologs responding with a similar pattern to a particular biological perturbation, calculation of expression correlation of orthologs in a single dataset cannot satisfactorily represent the expression conservation level.

### Major condition-specific changes in expression between *S. bayanus* and *S. cerevisiae*

Using an expression compendium, the global LNS measures general expression change between species but may not be sensitive to changes in condition-specific expression patterns in response to specific environmental or genetic perturbations. For instance, a gene may be expressed at a basal level in one condition, but be strongly activated relative to other genes in a second condition. For example, the effect of divergence of Ste12 binding sites on alpha factor gene expression response was only detected in an alpha factor dataset and not in a larger expression compendium [25], likely because there is minimal alpha factor signaling under conditions where alpha factor is absent. Since we anticipate that genes highly conserved in expression response in one condition could diverge significantly in another condition, so we investigated expression conservation using a dataset-specific approach. In order to more sensitively examine condition-specific gene expression conservation, we calculated the LNS for individual datasets or small, related groups of datasets similar in size and perturbation and examined the behavior of different functional groups (Table 2). This measure, which we call condition-specific LNS (Additional file 4: Table S3), is still independent of the timing of perturbation response in the two species, but provides a condition-specific measure of expression divergence, enabling us to more sensitively detect divergence that is specific to a particular condition.

**Table 2:** **Paired datasets of *S. bayanus *and *S. cerevisiae *for condition-specific LNS calculation**

***S. bayanus *dataset**	***S. cerevisiae *dataset**	**Number of diverged genes (lower than average of randomization, cutoff)**	***P *-value**	**Median LNS**
**MMS**	[51]	**1498 (−0.02515)**	**1.67E-153**	**0.2348**
**Heat shock**	[52]	**1032 (−0.00467)**	**4.25E-271**	**0.2259**
**Sorbitol**	[52]	**1330 (0.00561)**	**3.35E-163**	**0.1678**
**Uracil**	[53]	**1590 (0.001)**	**5.75443E-82**	**0.1031**
**Rapamycin**	[54]	**1896 (−0.0101)**	**1.63545E-33**	**0.07464**
**Alpha factor**	[55]	**1438 (−0.006)**	**5.5186E-102**	**0.0848**
**Sporulation**	[56]	**922 (0.0038)**	**3.7792E-288**	**0.08923**
**Cell cycle**	[57]	**1124 (0.0047)**	**1.7278E-226**	**0.09546**
**Diauxic shift**	[58]	**947 (0.00656)**	**1.1322E-257**	**0.3741**

We permuted gene-gene correlations to simulate the LNS distribution of a randomized network. The numbers of genes below the average LNS for these random networks were counted. We also calculated the *p-*value by randomizing the matches between orthologs to simulate non-conservation. The average LNS for each matched dataset pair was calculated.

**Figure 3:**
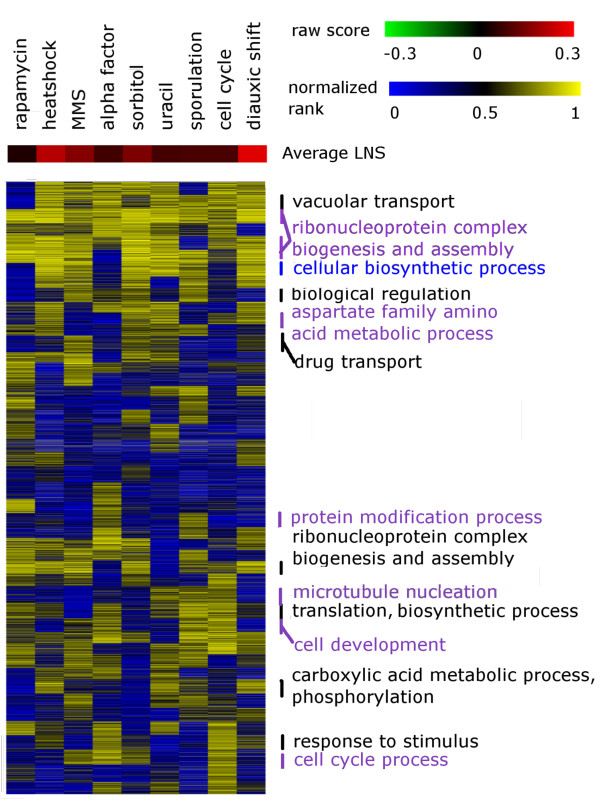
**Variation in expression conservation of genes of different functional groups under different perturbations.** The LNS for each gene for matched datasets of the two species was calculated. LNS scores were first k-means clustered and then arranged hierarchically by the centers of these clusters, and the scores presented by a heat map. GO biological process enrichment for each cluster was determined. Enriched terms with a Bonferonni-corrected *p* value lower than 0.01 are labeled. The expression patterns of ribosome-related genes are conserved upon most perturbations. However, the expression of many of the genes of other functions is only conserved under specific conditions. The range of LNS scores varies for different datasets. Datasets with large magnitude expression changes tend to have a greater LNS range; dataset size and quality also influence the range of LNS.

We found that, overall, orthologs exhibited some level of conservation in expression pattern regardless of the treatment, as expressed in the bias towards positive condition-specific LNS across matched datasets (Figure 3 and Table 2). However, individual experimental treatments and genetic perturbations demonstrated different patterns of expression conservation. For example, in the heat shock and diauxic shift treatments, genes on average showed high expression conservation (Figure 3 and Table 2). Other perturbations, for example, alpha factor treatment, uracil limitation, and rapamycin treatment, exhibited a relatively higher number of genes with divergent expression, although the majority of genes were still well-conserved in these datasets. The differences among the LNS distributions are most likely due to both the properties of the experimental treatment and the quality and size of the two datasets compared for each treatment. In order to normalize these effects and attempt to quantify the number of genes with divergent expression across matched datasets, we randomized the ortholog match for each dataset pair so as to simulate the situation of no conservation. In other words, to generate the randomized set, for all ortholog pairs, one member of the pair was matched with a random ortholog in the other species (with the random ortholog coming from some other orthologous pair). In general, around a quarter of the orthologs had a condition-specific LNS lower than the average LNS of randomized datasets. This indicates even very conserved biological responses elicit different gene expression consequences in the two species.

Genes of different functional categories showed differential levels of expression divergence under specific conditions. In general, genes of ribonucleoprotein complex biogenesis and assembly (a term which contains primarily genes involved in ribosome structure and assembly) showed highly conserved expression patterns regardless of the nature of the expression perturbation. Other categories of genes showed more specific patterns of conservation. For example, cell cycle genes were most conserved in the cell cycle dataset and alpha factor treatment. In datasets such as rapamycin or uracil treatment, these genes did not show specific conservation in their coexpression network. This result indicates that conclusions on gene expression conservation can be generic (e.g., ribosomal-related genes) or true under only very specifically defined conditions.

Condition-specific LNS identified mis-annotated genes in *S. bayanus* that were overlooked by the global LNS analysis. For example, *S. bayanus* gene *636.13* was matched to *CLB2* (*YPR119W*) in the initial annotation efforts by [8]. However, it has a low cell cycle LNS (−0.03) and this lack of expression conservation is evident by its shift in peak expression during the cell cycle (Additional file 3: Figure S3). *636.13* instead corresponds to *CLB5* based on both synteny and Blast alignment [44]. This change in gene expression was observable in the condition-specific LNS analysis of cell cycle synchronized cells because this alternate phase of expression changed the correlations with other periodically regulated genes. In the global dataset that consisted of primarily asynchronous cells, these phase specific correlations were not present.

**Figure 4:**
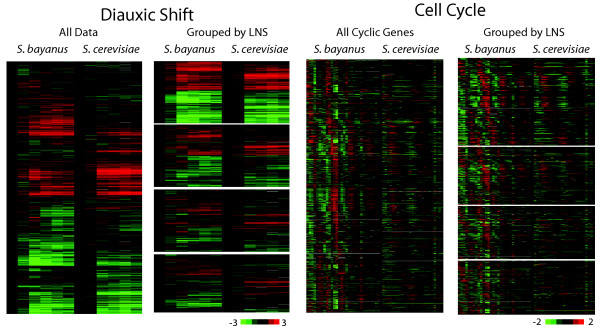
**Condition-specific LNS sorting of expression data into conserved and non-conserved patterns. A**. Expression data from the diauxic shift in both *S. bayanus* and *S. cerevisiae* were separately zero-transformed, and then aligned so that each horizontal line of data contains the expression of an orthologous pair of genes. The paired expression data were hierarchically clustered by uncentered Pearson correlation [59]. The data are presented by a heat map of log_2_ expression values. *S. cerevisiae* diauxic shift data were from [58]. **B**. The clustered data were partitioned by LNS score into quartiles, preserving the order of the genes in the original cluster. Genes with different expression between the species have low LNS scores. **C**. Cell cycle data from MATa cells synchronized by alpha factor arrest were mean centered. *S. cerevisiae* cell cycle data was obtained from [57]. For each species, the phase of the cell cycle was determined by Fourier transformation, and the top genes mapped to the phase were determined as cell cycle-regulated. Orthologs with either member of the ortholog pair determined as cell cycle-regulated are presented. The orthologs were arranged by the time of peak expression in *S. bayanus*.

We take two examples here to illustrate major changes in the expression response to environmental change between the two species. First, we quantized the expression data from the diauxic shift in *S. bayanus* and *S. cerevisiae*[58] based on their diauxic shift condition-specific LNS (Figure 4A-B). We observed that although the majority of the genes preserved their expression response upon diauxic shift, the lower quartile (by condition-specific LNS) of the orthologs displayed largely anti-correlated expression. Accordingly, 941 orthologs displayed a negative condition-specific LNS in diauxic shift. *S. bayanus* and *S. cerevisiae* have several observed differences in fermentation behavior [60-62], some of which could be associated with the changes in gene expression that we observe.

A similarly large fraction of divergent genes was observed for the paired cell cycle data. We identified 591 (in *S. bayanus*) and 626 (in *S. cerevisiae*) cell cycle-regulated genes whose one-to-one orthologs have data in the other species, making approximately the same proportion of genes cell cycle-regulated in both species. In total this represents 1106 unique genes, with 111 pairs of orthologous genes periodic in both *S. bayanus* and *S. cerevisiae* (*p* < 0.002, two tailed *t*-test). We also assessed the conservation of cell cycle specific gene expression using the 800 genes previously identified by a different dataset [55] as periodically expressed in synchrony with the cell cycle in *S. cerevisiae*, and observed that 226 of these orthologs were periodically expressed in synchronous cultures of *S. bayanus* (p<0.001). A large fraction of the periodically expressed genes were only cell cycle regulated in one of the species (Figure 4C-D): of the 1106 cell cycle regulated genes identified in either species, 258 had a cell cycle-specific LNS lower than 0, indicating significant change in their behavior over the cell cycle.

During the cell cycle, either phase changes or a presence of periodic expression in only one species could contribute to low LNS. We have recently correlated some of these changes in periodic gene expression with differential motif presence and nucleosome occupancy in these genes' promoters [10]. Some of the differences in timing could result from changes in the regulation of the cell cycle, but coherent cycling of protein levels could also be achieved even when gene expression is divergent. For example, if one member of a protein complex was periodically expressed in one species, another member of the same complex could be periodically expressed in the other species. This scenario would result in divergence of expression pattern even though the protein complex was periodically regulated through the availability of the cycling component [45,63,64]. Indeed we observe that although most cell cycle protein complexes retain cell cycle-regulated genes in both species, the identity of dynamic members differs between species (Additional file 3: Figure S4). These observations of expression divergence are not limited to the specific examples described above: all datasets have a fraction of divergently expressed genes despite the general trend of expression conservation observed over all data, underlining the importance of a dataset-specific measurement of expression conservation.

## Discussion

In this study, we employed a scalable measurement of expression conservation between species, Local Network Similarity, or LNS, to study the conservation of gene expression networks using large microarray data compendia from *S. bayanus* and *S. cerevisiae*. This unsupervised metric allowed us to quantify expression divergence between orthologs for datasets that are different in time-course sampling, or for species that have differential response kinetics to environmental perturbations. This distance metric scales the measurement of expression conservation between −1 and 1, with the null-hypothesis distribution centered at zero. Future research directions include extension of this metric to greater evolutionary distances and diverse data types beyond gene expression. We expect that the normalization inherent in the LNS metric will make it particularly robust for RNA-seq data in the face of the larger noise component that has been observed for genes with low expression levels [65-68].

Using the newly developed LNS metric, we found that patterns of expression-level divergence vary among different biological processes and functional groups. Certain central processes such as ribosome biogenesis are highly conserved on both the sequence and expression level. However, other functional groups involved in seemingly conserved behavior (e.g. cell cycle, diauxic shift) in fact include a significant fraction of orthologs whose expression diverges. This indicates that specific expression patterning of some genes is not critical to an organism’s response to environmental change. On the other hand, the overall conserved expression patterns between the two species might represent genes with key functional roles in responding to specific environmental changes.

One limitation of the current study is that we cannot determine to what degree sequence divergence explains overall expression network divergence between *S. bayanus* and *S. cerevisiae*. The selective forces acting on gene expression are as yet unclear and deriving a null model for gene expression evolution is a topic of active research (reviewed in [69-71]). Our observation of a bimodal distribution of LNS scores suggests that some genes could be evolving under selection for conserved expression patterns, while others may evolve more neutrally and thus show greater variance [72-74]. However, those genes that appear to be evolving neutrally could be simply not specifically perturbed in the conditions used for the available expression data. Addressing this challenge experimentally requires further collection of diverse expression datasets in genetically divergent strains within these species, as well as in other species across a range of evolutionary distances. Using such datasets, LNS can be used to delineate further how expression differences change with varying levels of sequence change.

## Conclusions

This study focuses on the response and expression profiling of *S. bayanus* under different perturbations. Complementary studies of regulatory networks, such as interrogation of transcription factor occupancy [75] and nucleosome positioning [10,76], will be useful to more fully characterize changes between species. Furthermore, while here we provide a prototype application of our network-based divergence measure (LNS) to gene expression, this approach should be extendable to other types of genome-wide data and can encompass diverse types of quantification of co-expression and/or network similarity. Extending our comparative approach to other groups of related species, such as *Candida* yeasts, *Drosophila* species, and mammals, could extend the observations made here. Since our experimental and analytical frameworks are agnostic to species and platform, they should be transferable to other systems. Such studies can be combined with sequence analysis to yield a more complete understanding of the mode of phenotype evolution and its relationship with sequence changes.

## Methods

### Pre-processing of *S. cerevisiae* and *S. bayanus* data

We assembled 303 arrays covering 46 datasets in *S. bayanus* [GEO: GSE16544] and 2569 arrays covering 125 datasets in *S. cerevisiae*. To allow reasonable comparison between datasets, we performed the following normalization steps. For each dataset, genes that are represented in less than 50% of the arrays were removed from this dataset, and missing values were estimated using KNNimpute with K = 10, Euclidean distance [77]. Finally, biological replicates are averaged, resulting in datasets with each gene followed by a vector representing its expression values in a series of arrays.

### Calculation of pair-wise correlation

For each dataset *i*, between gene pair expression vectors *j* and *k*, we calculated the correlation coefficient of their expression pattern:

(1) $$\rho \left(j_{i},k_{i}\right)=\frac{\mathrm{cov}\left(j_{i},k_{i}\right)}{\sigma_{j_{i}}\sigma_{k_{i}}}$$

To allow comparison between datasets, we Fisher’s z-transformed these correlation values [78]:

(2) $$z\left(j_{i},k_{i}\right)=\frac{1}{2}\ln\frac{1+\rho \left(j_{i},k_{i}\right)}{1-\rho \left(j_{i},k_{i}\right)}$$

For each dataset, these z values were then normalized to *Z’~N(0,1)*. We define the connection weights *z(j,k)* between any two genes *j* and *k* in a species as the average of the normalized *z* values over all datasets. This forms a pair-wise connection weight matrix for each species.

### Calculation of local network similarity (LNS) as a measurement of expression divergence

Connection weights between a specific gene *j* to all others form a vector *W(j)* = (*z(1,j), z(2,j)…z(N,j))*, where N is the total number of matched orthologs. *LNSj,j’* is defined as the correlation between the matched vectors (by orthology) of the two species, quantifying the conservation of the overall expression pattern of gene *j* (with its ortholog being j’) (Figure 1):

(3) $$\mathrm{LN}S_{j,j'}=\frac{\mathrm{cov}\left(W\left(j\right),W\left(j'\right)\right)}{\sigma_{W\left(j\right)}\sigma_{W\left(j'\right)}}$$

To assess the conservation level of orthologs upon specific biological perturbation (condition-specific LNS), we manually selected 10 datasets in *S. bayanus* that have matched time-course data in *S. cerevisiae*. For each data pair, we define the connection weight as the standard normalized value of formula (2), followed by the calculation of condition-specific LNS according to formula (3) (Figure 3).

### Correlation between sequence divergence and LNS

Measures of sequence divergence were used as in [26], including dN, dN/dS, Ka, and Ka/Ks as previously calculated [7,79]. A normal distribution was obtained by log_2_ transforming the data, mean subtracting it, and normalizing by the standard deviation. Correlation was calculated using the Pearson correlation.

### Clustering of condition-specific LNS and functional enrichment analysis

We determined the number of clusters in the condition-specific LNS matrix according to [80], resulting in 48 clusters. The enrichment of genes in each cluster was calculated through the program GOTermFinder [81].

### Identification of cell cycle regulated expression

We acquired *S. cerevisiae* cell cycle data from [57]. The following steps of identification of cell cycle regulated genes were applied to each species. For each gene in the cell cycle data, the expression values were centered so that the average over the time course equals to 0. A Fourier transformation was applied to the dataset of individual species so as to identify the period of cell cycle [55]. In *S. bayanus* the top 613 genes mapping to the phase were chosen as cell cycle regulated; and 785 genes in *S. cerevisiae* were chosen. This corresponds to 601 and 644 genes in *S. bayanus* and *S. cerevisiae* having one-to-one orthologs in the other species respectively. Among them, 591 (*S. bayanus*) and 626 (*S. cerevisiae*) have expression data in the other species, with 111 pairs overlapping.

## Competing interests

The authors declare they have no competing interests.

## Authors’ contributions

YF developed the LNS metric, calculated global LNS, and correlated it to sequence features. MJD and AAC analyzed LNS data and sequence similarity measures. YF calculated and clustered condition-specific LNS. All authors conceived the study, participated in interpretation of the results, and wrote the paper. All authors read and approved the final manuscript.

## Supplementary Material

Additional file 1: Table S1 Global between-species LNS for *S. cerevisiae* and *S. bayanus*. Table S1 presents the global LNS for all ortholog pairs in *S. cerevisiae* and *S. bayanus.* Click here for file

Additional file 2: Table S2 Within-species LNS for *S. cerevisiae*. Table S2 presents the LNS calculated within *S. cerevisiae* by subsampling the dataset. Click here for file

Additional file 3: Figure S1 Correlation of LNS with sequence features. The r value calculated by Pearson correlation between the LNS and the conservation of the indicated sequence is indicated. The p-value is calculated by using a test randomizing sequence similarity and LNS matches. Because the presence and conservation of a TATA box is a categorical value, that correlation is calculated using the point-biserial correlation. **Figure S2.** Correlation of LNS and various measures of sequence divergence. Expression similarity between orthologs, quantified as local network similarity (LNS), versus sequence similarity. LNS is correlated with indicated sequence divergence metrics with Pearson correlations of -0.215 (p<2.2e-16) for dN/dS, -0.238 (p<2.2e-16) for adjusted dN/dS, -0.222 (p<2.2e-16) for Ka, and -0.169 (p = 1.70e-14) for Ks/Ks. Black line is loess smoothed curve line and red line is running average of LNS summed over 100-gene windows. **Figure S3.** Cell-cycle-specific LNS identifies mis-annotation of *S. bayanus* gene *636.13*. Among the central cell cycle-regulated genes, *CLB2 (636.13)* had a particularly low condition-specific LNS (-0.03). The expression pattern of this gene was shifted during the cell cycle. Analysis of sequence and synteny reveals that *636.13* is the ortholog of *CLB5* rather than *CLB2*. **Figure S4.** Orthologous complexes involving cell-cycle regulated genes. *S. cerevisiae* MIPS protein complexes curated by (de Lichtenberg, Jensen et al. 2005) with more than 4 orthologous genes were included if at least one of the genes is cell-cycle regulated (as determined in the main text) in either species. Nodes depict genes and lines connecting them depict physical interactions. Cell cycle regulated genes are highlighted in red: A. *S. bayanus* B. *S. cerevisiae*. For complexes that have cell cycle regulated genes in both species, the dynamic member frequently switches. Click here for file

Additional file 4: Table S3 Condition-specific between-species LNS for *S. cerevisiae* and *S. bayanus*. Table S3. presents the condition-specific LNS for each gene. Click here for file
