# Supplementary material for: Comparative gene expression between two yeast species
Source: BMC Genomics. 2013 Jan 16;14:33. doi: 10.1186/1471-2164-14-33 (PMC3556494; doi:10.1186/1471-2164-14-33)
Supplement: Additional file 3: Figure S1 — Correlation of LNS with sequence features. The r value calculated by Pearson correlation between the LNS and the conservation of the indicated sequence is indicated. The p-value is calculated by using a test randomizing sequence similarity and LNS matches. Because the presence and conservation of a TATA box is a categorical value, that correlation is calculated using the point-biserial correlation. Figure S2. Correlation of LNS and various measures of sequence divergence. Expression similarity between orthologs, quantified as local network similarity (LNS), versus sequence similarity. LNS is correlated with indicated sequence divergence metrics with Pearson correlations of -0.215 (p<2.2e-16) for dN/dS, -0.238 (p<2.2e-16) for adjusted dN/dS, -0.222 (p<2.2e-16) for Ka, and -0.169 (p = 1.70e-14) for Ks/Ks. Black line is loess smoothed curve line and red line is running average of LNS summed over 100-gene windows. Figure S3. Cell-cycle-specific LNS identifies mis-annotation of S. bayanus gene 636.13. Among the central cell cycle-regulated genes, CLB2 (636.13) had a particularly low condition-specific LNS (-0.03). The expression pattern of this gene was shifted during the cell cycle. Analysis of sequence and synteny reveals that 636.13 is the ortholog of CLB5 rather than CLB2. Figure S4. Orthologous complexes involving cell-cycle regulated genes. S. cerevisiae MIPS protein complexes curated by (de Lichtenberg, Jensen et al. 2005) with more than 4 orthologous genes were included if at least one of the genes is cell-cycle regulated (as determined in the main text) in either species. Nodes depict genes and lines connecting them depict physical interactions. Cell cycle regulated genes are highlighted in red: A. S. bayanus B. S. cerevisiae. For complexes that have cell cycle regulated genes in both species, the dynamic member frequently switches. [file 1471-2164-14-33-S3.doc]

**
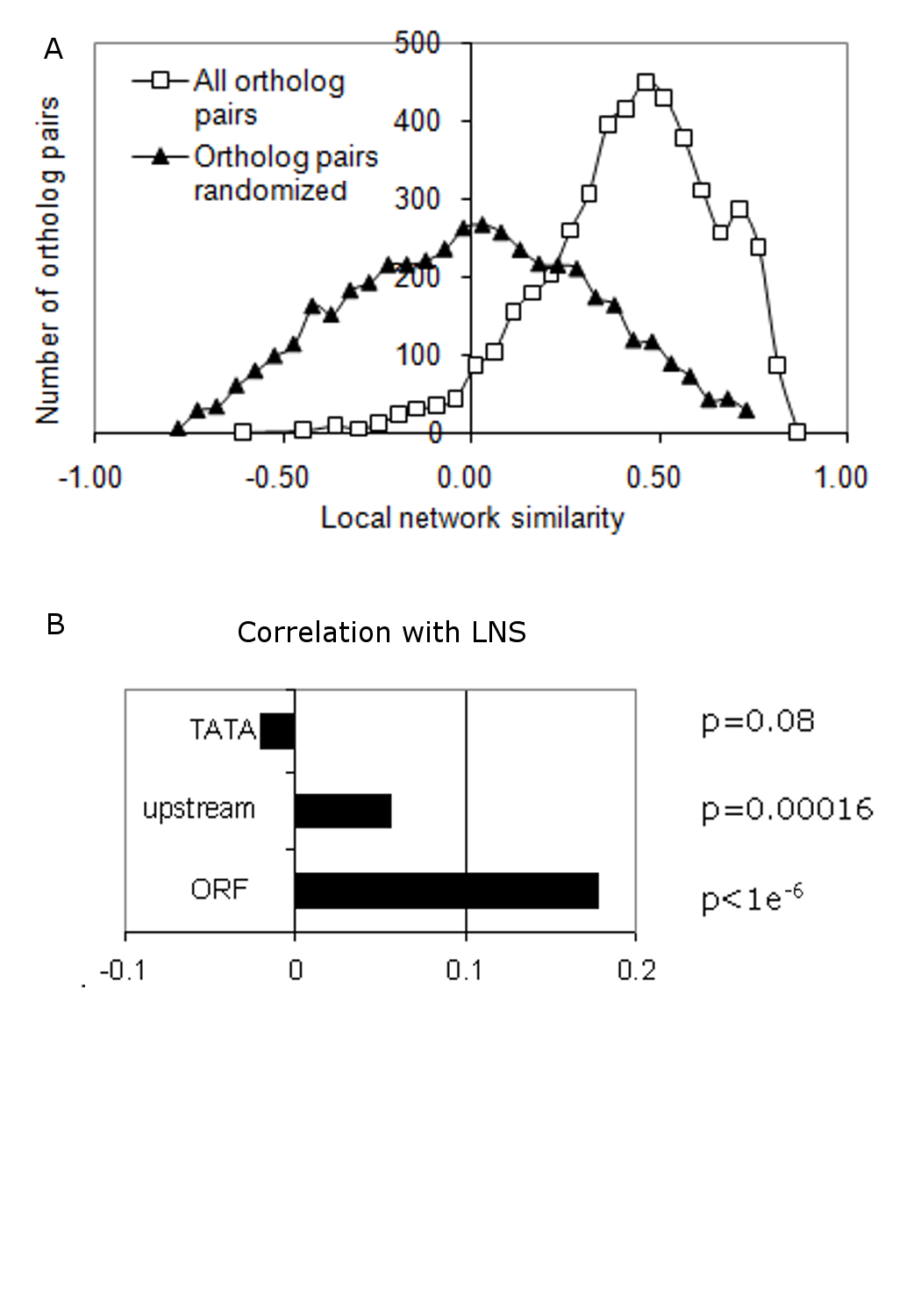
**

**
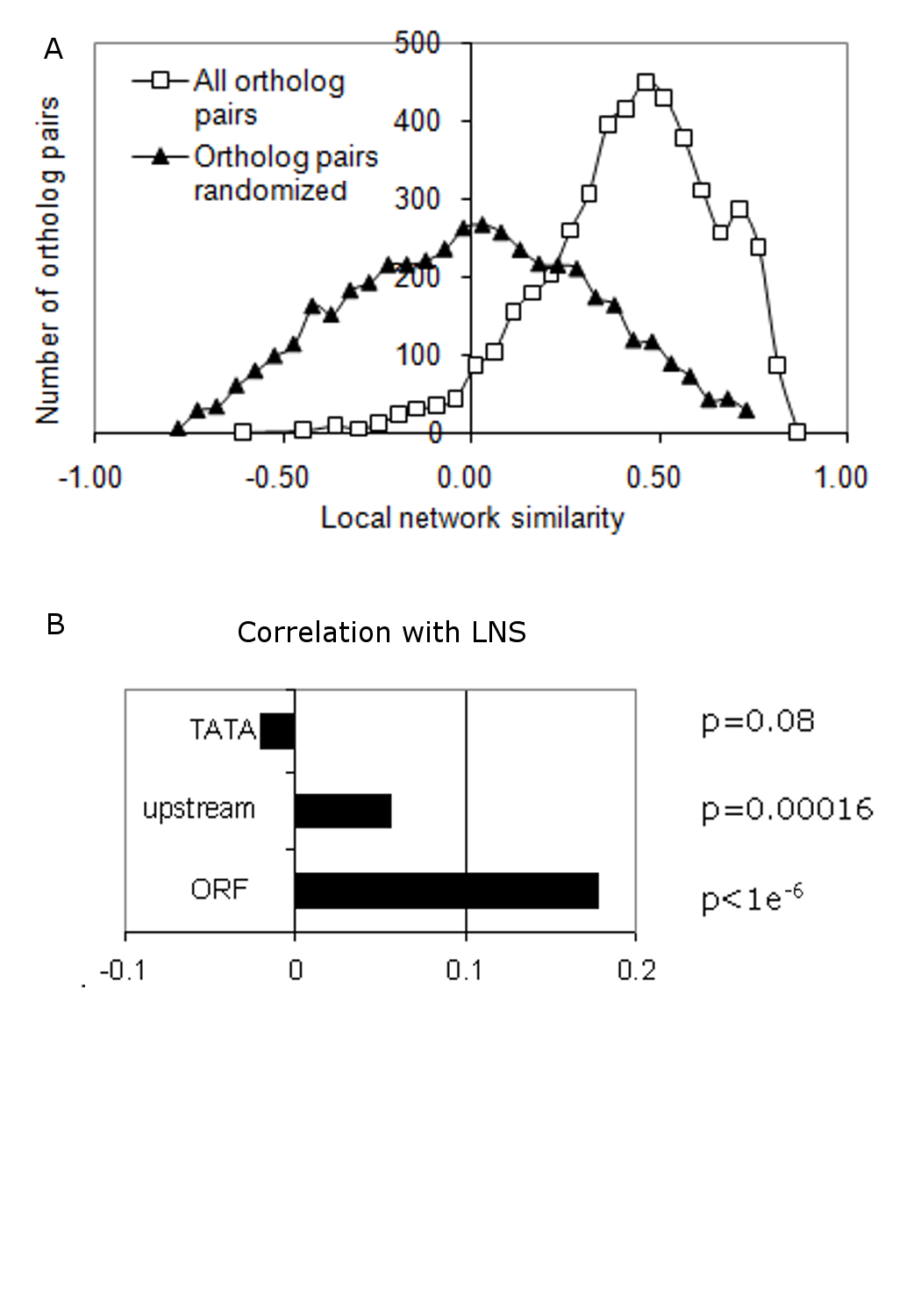
**

**Figure S1 - Correlation of LNS with sequence features.**

The r value calculated by Pearson correlation between the LNS and the conservation of the indicated sequence is indicated. The p-value is calculated by using a test randomizing sequence similarity and LNS matches. Because the presence and conservation of a TATA box is a categorical value, that correlation is calculated using the point-biserial correlation.

**Figure S2 -** **Correlation of LNS and various measures of sequence divergence.**

Expression similarity between orthologs, quantified as local network similarity (LNS), versus sequence similarity. LNS is correlated with indicated sequence divergence metrics with Pearson correlations of -0.215 (p<2.2e-16) for dN/dS, -0.238 (p<2.2e-16) for adjusted dN/dS, -0.222 (p<2.2e-16) for Ka, and -0.169 (p = 1.70e-14) for Ks/Ks. Black line is loess smoothed curve line and red line is running average of LNS summed over 100-gene windows.

**
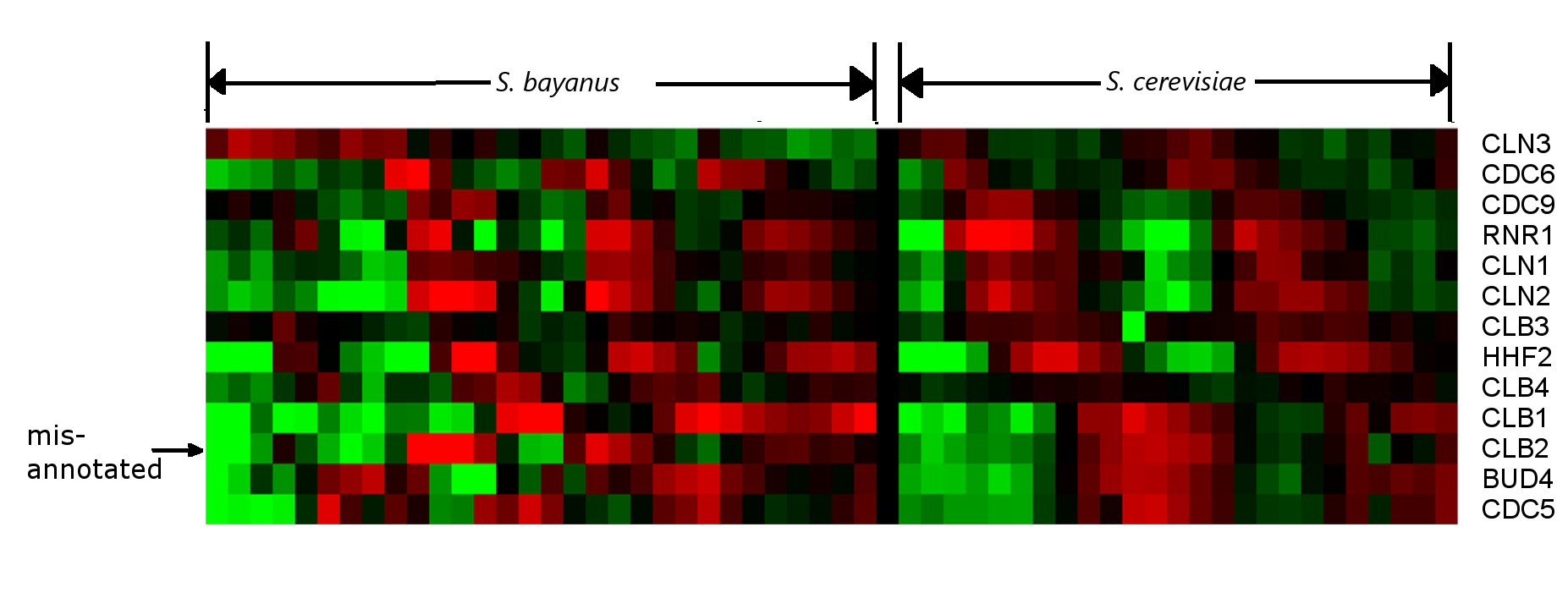
Figure S3 - Cell-cycle-specific LNS identifies mis-annotation of *S. bayanus* gene *636.13*.**

Among the central cell cycle-regulated genes, *CLB2 (636.13)* had a particularly low condition-specific LNS (-0.03). The expression pattern of this gene was shifted during the cell cycle. Analysis of sequence and synteny reveals that *636.13* is the ortholog of *CLB5* rather than *CLB2*.

**
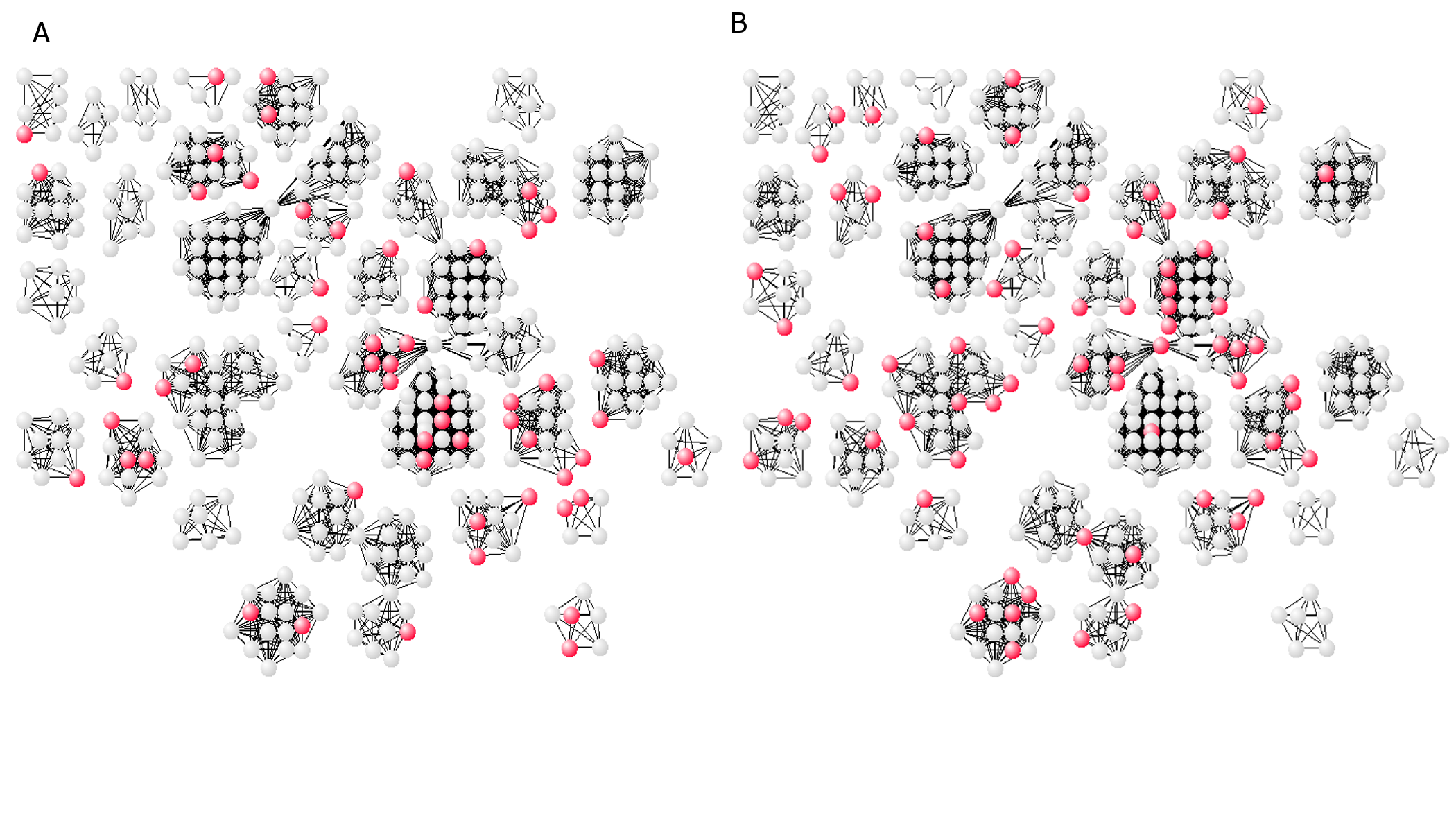
**

**Figure S4 - Orthologous complexes involving cell-cycle regulated genes.**

*S. cerevisiae* MIPS protein complexes curated by (de Lichtenberg, Jensen et al. 2005) with more than 4 orthologous genes were included if at least one of the genes is cell-cycle regulated (as determined in the main text) in either species. Nodes depict genes and lines connecting them depict physical interactions. Cell cycle regulated genes are highlighted in red: A. *S. bayanus* B. *S. cerevisiae*. For complexes that have cell cycle regulated genes in both species, the dynamic member frequently switches.
